# Structural and Psychological Empowerment in Explaining Job Satisfaction and Burnout in Nurses: A Two-Level Investigation

**DOI:** 10.1155/2023/9958842

**Published:** 2023-06-24

**Authors:** Agnieszka Orlowska, Mariola Laguna

**Affiliations:** The John Paul II Catholic University of Lublin, Institute of Psychology, Lublin, Poland

## Abstract

**Aim:**

The aim of the study was to test a two-level model of the relationships between structural empowerment in a hospital department and job satisfaction and burnout in nurses. We tested whether psychological empowerment is a mediator of these relationships.

**Background:**

We drew on empowerment theory to examine whether psychological empowerment mediates the association between organizational-level structural empowerment and nurses' job satisfaction and burnout at the individual level. The proposed two-level model takes into account the effect of both contextual and individual factors on individual nurse's job satisfaction and burnout.

**Methods:**

The study was conducted in 29 public hospital departments and included 309 participants employed as nurses or midwives. To analyze the results, we applied multilevel modeling and cross-level mediation analysis, with nurses as Level 1 and hospital departments as Level 2.

**Results:**

Structural empowerment at the hospital department level is positively related to nurses' individual sense of competence and autonomy, namely, to their psychological empowerment. Structural empowerment is also positively related to job satisfaction and negatively related to burnout in nurses. Psychological empowerment is a mediator between structural empowerment and nurses' job satisfaction as well as two dimensions of burnout: exhaustion and disengagement from work.

**Conclusions:**

These findings suggest that psychological empowerment is an underlying mechanism that may explain why structural empowerment in the hospital department is positively related to job satisfaction and negatively related to burnout in nurses. This has implications for theory by extending the multilevel nomological network of the constructs and for management practice by highlighting the role of structural empowerment for work design in public health institutions. *Implications for Nursing Management*. The results indicate that structural and psychological empowerment can play a significant role in creating supportive workplace conditions in hospitals. Organizing nurses' work in a way that empowers them promotes their sense of competence and autonomy, which in turn promotes their job satisfaction and reduces burnout.

## 1. Introduction

The nursing shortage is a global problem [1] caused, among other factors, by staff turnover, resulting from unsatisfactory work environments [1, 2] and burnout, which is prevalent among nurses worldwide [3]. Nurses are expected to provide patient care with empathy and patience, while working in a highly stressful environment, with few resources and excessive workloads, thus requiring from nurses to balance multiple pressures [4]. Given the nursing shortage, special attention should be paid to supportive work conditions that provide high job satisfaction and prevent burnout. Burnout, a great threat to healthcare professionals, especially to nurses [5], develops as a result of excessive and unbalanced workplace demands and is expressed in exhaustion and disengagement from work [6]. Exhaustion is an effect of chronic tension caused by job demands, and nursing is one of the most exhausting professions as a result of various challenges in professional practice [4]. Disengagement from work is an attitude of withdrawal from patients, colleagues, and the whole context related to work [6]. Conversely, nurses' job satisfaction has been shown to be associated with a variety of positive outcomes, including higher task performance and retention, lower absenteeism, and lower turnover (see for review [7]). Therefore, a better understanding of the organizational practices which may lead to higher job satisfaction, lower exhaustion, and lower disengagement from work among nurses' is important for healthcare management.

Some personal and organizational antecedents of burnout and job satisfaction in healthcare professionals have already been uncovered (for a review, see [4, 8]). However, the mechanisms that explain how organizational factors relate to individual outcomes in employees await further investigation. Human resource management strategies based on the empowerment theory [9, 10] have been observed to bring positive organizational outcomes and to prevent negative outcomes in a variety of organizations (e.g., [11]), including healthcare (e.g., [12]). There is also already evidence that empowerment is positively related to job satisfaction and negatively related to burnout in nurses (for reviews and meta-analyses, see [1, 13, 14]). However, most research to date, with rare exceptions (e.g., [10, 15]), has treated employee empowerment as an individual-level phenomenon. Too little attention has been paid to the fact that the relationships between organizational-level and individual-level constructs have a natural multilevel structure, i.e., empowerment strategies are implemented in healthcare organizations (i.e., at the organizational level) and their effects on job satisfaction or burnout are observed in individual employees. The multilevel approach may shed new light on the mechanisms linking organizational empowerment with its psychological consequences. Therefore, it remains to be investigated whether the results from single-level studies that dominate to date and were synthesized in reviews and meta-analyses (e.g., [1, 13, 14]) can be replicated in studies using multilevel designs.

To fill this gap, in the present study, we propose and test a theoretical model that postulates multilevel relationships between nurses' shared perceptions of empowerment at the unit level and nurses' individual-level job satisfaction and burnout. We also propose that psychological empowerment is an underlying mechanism that may explain these relationships. Given the inconsistencies in previous studies testing similar mediation mechanisms (e.g., [1, 16]), new multilevel evidence may shed light on this issue. The results of the study can serve as a basis for recommendations regarding the organization of nurses' work in hospital departments.

## 2. Theoretical Background

Empowerment theory has been widely applied in analyses of nurses' work [7, 9, 10, 17, 18]. It proposes a management strategy based on the implementation of systemic and consistent human resource management practices in the workplace that increase nurses' commitment by creating an atmosphere of openness and trust to improve their outcomes (e.g., [19]). The empowerment strategy obliges the organization's management to provide nurses with greater power and autonomy in performing their duties and making decisions related to their work. Empowerment enables the proper performance of duties and the pursuit of the hospital's interests; it also increases work motivation [20]. This happens when nurses gain more control over their work and want to participate in decisions that affect them [21].

In the organizational context, the term *empowerment* is used with reference to two perspectives [15]: structural (the organizational-level construct) and psychological (the individual-level construct). Structural empowerment is a set of purposeful management actions and polices that provide power, control, and authority to subordinates [15]. These practices are reflected in nurses' shared perceptions of structural empowerment in their work units, such as hospital departments [15]. These management practices aim to empower employees, that is, to make them stronger and more independent by creating an organizational context that leads to state empowerment or empowerment at the psychological level. *Psychological empowerment* refers to employees' sense of competence and autonomy [1, 22]. Consequently, organizational-level structural empowerment is expected to have an impact on individual-level psychological empowerment.

According to Kanter's [23] theory, structural empowerment is reflected in six dimensions [24]. (1) Access to opportunity is defined as with access to challenges, rewards, and opportunities for improvement, as well as full use of the employee's skills and knowledge. (2) Access to information means having knowledge about the values held in the organization and about the goals and policies of management and using this knowledge. (3) Access to support includes feedback from supervisors and peers, as well as advice on how to solve problems. (4) Access to resources is defined as the time needed to perform certain actions and access to the materials, equipment, and money needed to do the job. (5) Formal power is associated with job characteristics: flexibility, the employee's creative contribution to the achievement of the organization's goals, and the extent to which individual employees are authorized to make decisions. (6) Informal power refers to efficient communication between employees and management, cooperation in an atmosphere of friendship, and the employee's sense of being useful to colleagues and supervisors who look to him or her for support in problematic situations.

Creating empowering work conditions is considered an important organizational strategy that contributes to psychological empowerment and ultimately leads to positive work behaviors and attitudes [12, 25, 26]. Nurses who work for a particular organization and are provided with the information, support, and resources they need to do their jobs, as well as ongoing opportunities for development, may experience a greater sense of autonomy and job self-efficacy, which are essential to psychological empowerment [27, 28]. Psychologically empowered employees perform their work with a sense of control over what they do and are engaged in their work [11], and their productivity increases, and so does the effectiveness of their actions [27]. Of interest to this research, psychological empowerment improves job satisfaction (for review, see [13]) and reduces burnout in nurses (for review, see [4]) and consequently reduces nurse turnover [1]. However, the cross-level mechanisms explaining these effects (i.e., linking organizational and individual-level constructs) remain to be explored.

## 3. The Present Study

According to the theories of Kanter [23] and Spreitzer [28], workplace behaviors are determined by the social structures of the workplace. Thus, nurses' shared perceptions of structural empowerment in their work unit (e.g., hospital department) are expected to reflect organizational-level management practices specific to that unit, which in turn are reflected in the individual-level attitudes and behaviors of nurses. Psychological empowerment is thus a logical outcome of structural empowerment, and powerless individuals may be more susceptible to burnout and reduced job satisfaction [20, 29].

**Figure 1:**
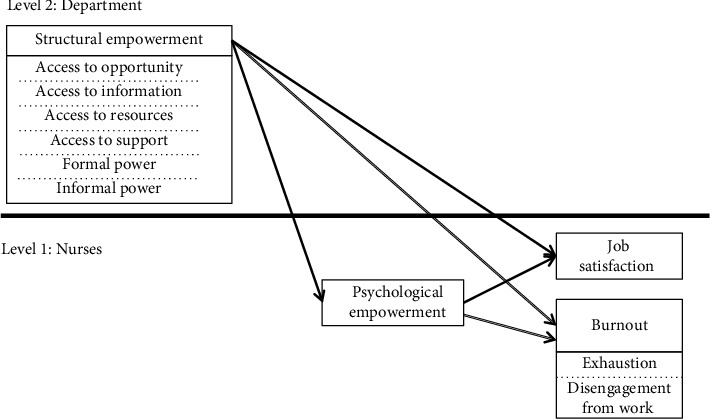
Conceptual two-level model of relationships between structural empowerment, psychological empowerment, job satisfaction, exhaustion, and disengagement from work.

Based on these premises, we propose a two-level model (Figure 1) in which psychological empowerment is an underlying mechanism that explains why structural empowerment at the organizational level is related to job satisfaction and burnout at the individual (i.e., nurse) level. Because nurses are employed in specific hospital departments and because analyzing their work without considering the differentiation of managerial strategies at the department level seems to provide an incomplete picture of the analyzed relationships, our model explains cross-level links between constructs, with nurse at Level 1 and department at Level 2. Consequently, our research answers the research problem whether psychological empowerment is a mediator between structural empowerment at the organizational level and (1) job satisfaction and two dimensions of burnout: (2) exhaustion and (3) disengagement from work at the individual level. Below, we explain the cross-level mediations included in our model.

To examine the cross-level mechanism linking structural empowerment to job satisfaction through psychological empowerment, we first consider existing evidence on the individual relationships among these three constructs, which supports our model. Research has shown that structural empowerment is positively related to psychological empowerment [13], which culminates in positive outcomes at work [12, 25, 27, 29, 30]. A positive relationship between psychological empowerment and job satisfaction was found in many studies and supported by the results of systematic reviews and meta-analyses [1, 7, 13]. These consistent conclusions concern specific relationships between these three variables, while studies testing a mediation mechanism itself provide mixed evidence. The model postulating a mediating role of psychological empowerment between structural empowerment and job satisfaction was supported in a Canadian study [31]. However, longitudinal studies [32] and studies of Taiwanese nurses [33] did not support this relationship. Furthermore, a meta-analysis [1] found no empirical support for such mediation. Nevertheless, these studies were based on single-level data, and the analyses mainly included the total score of structural empowerment rather than its dimensions. Therefore, to address the discrepancies in previous findings and to answer the call for further studies [1], we propose a multilevel approach and formulate the following hypotheses:

 
*Hypothesis 1*. Nurses' psychological empowerment is a mediator between structural empowerment at the department level and nurses' job satisfaction.
   Similarly, when looking for specific relationships between constructs included in the second mechanism explaining burnout, research shows negative relationships between psychological empowerment and job burnout (e.g., [34]). Research on nurse burnout indicates that psychological empowerment acts as a buffer against emotional exhaustion and disengagement from work [35, 36]. However, there are inconsistencies in the existing evidence regarding the mediating role of psychological empowerment between structural empowerment and burnout. Some studies support this mediation mechanism [3, 29, 35], while others do not [37]. To reconcile these discrepancies resulting from single-level research, we consider cross-level relationships. In our multilevel model, we postulate a mediating role of nurses' psychological empowerment between structural empowerment at the department level and nurses' burnout, which is reflected in the hypotheses regarding the two dimensions of burnout.
 
*Hypothesis 2*. Nurses' psychological empowerment is a mediator between structural empowerment at the department level and nurses' exhaustion.
 
*Hypothesis 3*. Nurses' psychological empowerment is a mediator between structural empowerment at the department level and nurses' disengagement from work.

Studies have shown that demographic characteristics (e.g., age, sex, and work experience) are significant predictors of structural empowerment, nurses' job satisfaction, and burnout [7, 14, 35, 38]. Therefore, in our analyses, we control for age, sex, nursing work experience, weekly working time, and extra work taken on.

## 4. Materials and Methods

### 4.1. Procedure

We obtained consent for the research from the hospital's management and the head nurse. Participants were informed of the purpose of the study; they were also informed that their participation was voluntary and anonymous and that they could withdraw at any time. They were asked to complete the paper-and-pencil questionnaires at a convenient time and place and return them to the special boxes provided in the hospital departments. To minimize the incidence of common method bias [39], we divided the questionnaire into sections—items related to different constructs were presented on separate pages.

The sampling criterion was employment as a nurse or midwife. Data were collected in 2019 in Poland in 29 public hospital departments (e.g., obstetrics and gynecology, surgical, orthopedic, and internal diseases departments).

### 4.2. Participants

The study included 309 respondents employed as hospital nurses (270, 87% of the sample) or midwives (39, 13%): 292 women and 17 men. Their age ranged from 21 to 65 years (*M* = 43.59, SD = 11.16). The majority of respondents had a master's (115) or bachelor's degree (111), 81 had secondary education, and two respondents had vocational education. Most of them (296) were employed full time. For the majority of respondents, this employment was permanent (270), 31 respondents had a fixed-term employment contract, and 7 worked on the basis of an order contract or other kind of contract. Their work experience in the profession ranged from one year to 44 years (*M* = 19.65, SD = 12.71), and the average length of employment with the current employer was over 16 years (*M* = 16.51, SD = 12.47). Participants worked an average of 41 hours per week (SD = 5.62); most of them worked both day and night shifts (239 respondents); 68 respondents (22%) reported working only day shifts, and one respondent reported working only night shifts. Seventy-three respondents had additional paid work (e.g., in an outpatient clinic and home care).

### 4.3. Measures

Structural empowerment was measured using the Conditions of Work Effectiveness Questionnaire II (CWEQ-II; [16]), as adapted into Polish by Orłowska and Łaguna [40]. The measure provides a total score for structural empowerment, as well as scores for its six dimensions. Each scale consists of three items, with the exception of the Informal Power scale, which consists of four items. The Access to Opportunity scale begins with the question: “How much of each kind of opportunity do you have in your present job?,” followed by a list of opportunities (e.g., “Challenging work”). The Access to Information scale begins with the question: “How much access to information do you have in your present job?,” followed by a list of types of information (e.g., “The goals of top management”). The Access to Support scale begins with the following question: “How much access to support do you have in your present job?,” followed by a list of types of support (e.g., “Specific information about things you do well”). The Access to Resources scale begins with the question: “How much access to resources do you have in your present job?,” followed by a list of resources (e.g., “Time available to do necessary paperwork”). The Formal Power scale begins with “In my work setting/job,” followed by workplace characteristics (e.g., “The rewards for innovation on the job”). The Informal Power scale begins with the question: “How much opportunity do you have for these activities in your present job?,” followed by a list of opportunities (e.g., “Collaborating on patient care with physicians”). Responses are indicated on a 5-point scale, with anchors labeled according to the content of a given item (e.g., 1 = *none* to 5 = *a lot*). The reliability of the scales, as assessed by Cronbach's *α*, ranged from 0.79 to 0.93 (see Table 1).

To measure *psychological empowerment*, we used the Psychological Empowerment Instrument [28], adapted into Polish by Orłowska and Łaguna [41]. The questionnaire consists of 12 items (e.g., “My impact on what happens in my department is large”) that make up the total score. The items are rated on a 7-point scale (1 = *very strongly disagree* to 7 = *very strongly agree*). The reliability is *α* = 0.89.

To assess the level of *job satisfaction*, we used the Job Satisfaction Scale [42]. It is constructed in the same way as the Satisfaction with Life Scale [43] and consists of 5 items designed to assess the cognitive aspect of general job satisfaction (e.g., “In many ways, my work is close to my ideal”). The items are rated on a 7-point scale (1 = *strongly disagree* to 7 = *strongly agree*). The reliability is *α* = 0.86.

To measure the level of *burnout*, we used the Oldenburg Burnout Inventory [44], adapted into Polish by Baka and Basińska [45]. The inventory consists of 16 items forming two scales, of 8 items each: Exhaustion (e.g., “I can tolerate the pressure of my work very well,” reverse scored) and Disengagement from Work (e.g., “It happens more and more often that I talk about my work in a negative way”). The items are rated on a 4-point scale (1 = *strongly disagree* to 4 = *strongly agree*). Reliability is *α* = 0.77 for Exhaustion and *α* = 0.61 for Disengagement.

### 4.4. Data Analysis

Before moving on to hypothesis testing, we conducted preliminary analyses. First, because all constructs were measured with self-report instruments, we tested for potential common method bias using Harman's single-factor test [39]. Second, we examined descriptive statistics and intercorrelations between study variables. Third, we tested the variance of the dependent variables at both levels (see next section). Next, we analyzed the role of the control variables: age, sex, work experience in the nursing profession, weekly working time, and extra work taken on. If a given control variable was a statistically significant predictor, we included it in further analyses.

The data collected had a multilevel structure, with nurses as Level 1 and hospital department as Level 2. Department-level data were aggregated, that is, we calculated mean scores for the nurses working in a particular department. In the analyses, we applied multilevel modeling [46] using HLM7 software. We tested models explaining (1) job satisfaction and two dimensions of burnout: (2) exhaustion and (3) disengagement from work, including—in separate models—the global score of structural empowerment (Model 1) and its six dimensions (Model 2) as predictors (see Table 2). In accordance with the recommendations on centering [46], Level-1 continuous variables were group-mean centered, while Level-2 variables were first standardized and then entered uncentered. Categorical variables were entered uncentered at both levels. When reporting the results, we report unstandardized *γ* regression coefficients.

The final step was to test the mediation hypotheses using PRODCLIN [47]. This program allows the testing of cross-level mediation effects by computing confidence intervals (CI) for values from multilevel analyses with more accurate type I error indicators and greater power than other tests [47]. If the CI does not include zero, this indicates a statistically significant mediation effect.

## 5. Results

### 5.1. Preliminary Analyses

First, we used Harman's single-factor test [39] to determine whether the total variance of all variables (respective scales' items) extracted by one factor exceeded 50%. The exploratory factor analysis with one factor showed that this factor explained about 28% of their variance. This indicates that the collected data are free from common method bias.

Second, we examined descriptive statistics and correlations, which are presented in Table 1. The statistically significant positive relationships of structural and psychological empowerment with job satisfaction and their significant negative relationships with both dimensions of burnout provide preliminary support for the hypotheses. It should be noted, however, that for two dimensions of structural empowerment (i.e., formal power and informal power), the direction of the relationship with burnout was positive—not negative, as expected.

Third, before the main analyses, we tested the unconditional multilevel model, which allowed us to estimate the variance of the explained variables at each level. The variance for job satisfaction was 32.14 at the nurse level and 4.10 at the department level; for burnout, it was 13.29 and 2.23, and for disengagement, it was 10.46 and 0.01, respectively.

Next, we tested whether the control variables were statistically significant predictors of the explained variables. For job satisfaction, none of the control variables tested proved to be a statistically significant predictor (all *p*s > 0.05). For exhaustion, the only statistically significant predictor was age (*γ* = 0.07, SE = 0.03, *p*=0.039). In the case of disengagement from work, no statistically significant relationships were found for any of the control variables. Therefore, following the recommendation that only statistically significant predictors should be retained in a multilevel model [46], only age was included as a control variable to explain exhaustion in further analyses.

### 5.2. Multilevel Analysis of Direct Effects

To test the hypotheses, we first verified the direct relationships postulated in the model (see Figure 1). The results of the multilevel modeling (Table 2) showed that the total structural empowerment score was a statistically significant predictor of psychological empowerment (*γ* = 1.70, SE = 0.52, *p* = 0.003). No statistically significant relationships were found between the dimensions of structural empowerment and psychological empowerment.

The analyses explaining job satisfaction (Table 2) revealed its statistically significant positive relationships with psychological empowerment (*γ* = 0.28, SE = 0.02, *p* < 0.001) and with structural empowerment total score (Table 2). The analyses conducted for the individual dimensions of structural empowerment revealed that two dimensions, namely, access to resources (*γ* = 1.50, SE = 0.33, *p* < 0.001) and formal power (*γ* = 1.30, SE = 0.48, *p* = 0.014), were statistically significant positive predictors of nurses' job satisfaction.

Analyses concerning exhaustion revealed its statistically significant negative relationships to psychological empowerment (*γ* = −0.13, SE = 0.02, *p* < 0.001) and structural empowerment total score (*γ* = −0.11, SE = 0.05, *p* = 0.040). The analyses for the dimensions of structural empowerment (Table 2) indicated that access to opportunity (*γ* = 0.82, SE = 0.39, *p* = 0.046) and informal power (*γ* = 0.97, SE = 0.43, *p* = 0.033) were predictors of exhaustion, and both relationships were positive.

Analyses concerning disengagement from work showed negative associations with psychological empowerment (*γ* = −0.14, SE = 0.02, *p* < 0.001) and with structural empowerment total score (*γ* = −0.09, SE = 0.03, *p* = 0.002). None of the dimensions of structural empowerment were statistically significant predictors of work disengagement (Table 2).

### 5.3. Cross-Level Mediation

To test the hypotheses, we analyzed cross-level indirect effects. Using the coefficient values for direct effects from the multilevel analysis, we calculated CIs for mediation effects (Table 3). The results showed that psychological empowerment was a mediator between structural empowerment total score and job satisfaction, 95% CI [0.19, 0.79]. This supports Hypothesis 1. However, we found no statistically significant cross-level mediation effects for any of the dimensions of structural empowerment. Psychological empowerment was also a mediator between total structural empowerment score and exhaustion, 95% CI [−0.40, −0.08], supporting Hypothesis 2. Similarly, there was a mediation effect for work withdrawal, 95% CI [−0.42, −0.09], supporting Hypothesis 3. No significant cross-level mediation effects were found for the dimensions of structural empowerment (Table 3).

## 6. Discussion

In this study, we proposed a multilevel model postulating that psychological empowerment is an underlying mechanism that explains relationships between nurses' shared perceptions of structural empowerment practices in the hospital department and job satisfaction and burnout at the individual level in nurses. The empirical study in public hospital units in Poland aimed to determine the cross-level relationships of structural empowerment with (1) job satisfaction and the two dimensions of burnout: (2) exhaustion and (3) disengagement from work, with psychological empowerment as a mediator.

The importance of nurses' job satisfaction has been demonstrated in numerous studies, which have found that it is associated with a variety of positive outcomes, including lower turnover [7], which is important in the context of the nursing shortage [1, 2]. The results of this study show that psychological empowerment is a mediator between structural empowerment introduced at the hospital department level and individual job satisfaction of nurses. Nurses who are empowered through supportive management practices are more likely to believe in their ability to contribute meaningfully to the workplace, which in turn increases their job satisfaction, as psychological empowerment is an important internal incentive factor [1, 22]. Our findings shed new light on the inconsistencies found in the results of previous studies, which are mostly based on single-level investigations [1, 13, 31–33]. We also add to rare multilevel evidence (e.g., [10]) and show that organizational leadership that creates empowering work conditions creates supportive practice environments [10]. Furthermore, our findings suggest that two dimensions of structural empowerment, namely, access to opportunities and formal power, are direct predictors of nurses' job satisfaction. This extends evidence from previous studies supporting these relationships for the total score of structural empowerment [7, 33, 48]. Our multilevel analysis reveals that nurses experience higher levels of job satisfaction when they are provided with opportunities at the department level, such as adequate time for each task, materials, equipment, and financial resources necessary to perform professional duties. In addition, nurses experience higher job satisfaction when they share perception of formal power. This means that they feel that their work is seen as innovative and flexible and is valued by their managers and colleagues.

Our research findings also shed more light on the relationships between empowerment and burnout that have been demonstrated in previous studies [4, 14, 20, 35, 36]. Multilevel analysis revealed that empowering working conditions implemented in the department were associated with higher psychological empowerment of nurses, which in turn was associated with their lower burnout. Not only the total score but also the informal power and access to opportunities dimensions of structural empowerment were significant predictors of nurses' burnout. Our findings add new multilevel evidence to single-level studies that mostly focus on the total score of structural empowerment [3, 5]. Cross-level mediation was supported, and psychological empowerment emerged as an underlying mechanism explaining why structural empowerment is negatively related to nurses' exhaustion and disengagement from work. Although no statistically significant mediation effects were found for any of the dimensions of structural empowerment, multilevel modeling yielded several interesting findings regarding exhaustion. Significant predictors of exhaustion were age, access to opportunities, and informal power. As in other studies [35, 36], nurses' level of exhaustion increases with age. Interestingly, our analyses also show that nurses experience higher levels of exhaustion when the employer provides them with broad access to opportunities to improve their skills. It can be concluded that the employer's expectation to constantly improve professional skills, especially for nurses with many years of professional experience, may lead to increased exhaustion. Similarly, Cavus and Demir [35] found positive relationships between access to opportunities and one of the dimensions of burnout. Our results also suggest that higher levels of burnout may be experienced by nurses who are frequently called upon by colleagues and supervisors to help solve problems (informal power). The scope of a nurse's duties in a hospital is very broad [4] and requires collaboration with physicians and other hospital staff. Our findings suggest that this collaboration should be based on principles of reciprocity and partnership [24], and if collaboration with physicians increases nurses' workload, it may increase their exhaustion.

This study contributes to the literature in the following ways. First, it extends the multilevel nomological network of organizational-level structural empowerment and its individual-level outcomes among nurses, while shedding light on the psychological processes through which these relationships occur. Analyses include not only the total score of structural empowerment but also its six dimensions, providing a more nuanced picture of their relationships with job satisfaction and burnout. Second, this study advances the empowerment literature by suggesting that psychological empowerment may explain why empowering workplace conditions introduced in the hospital department make nurses more satisfied with their work and prevent them from burnout. Although previous evidence has shown that empowerment is positively related to job satisfaction and negatively related to burnout in nurses [1, 13, 14], this study adds value by attempting to unravel the cross-level mechanism behind it.

### 6.1. Limitations

The current study is not without limitations. The cross-sectional design limits the ability to make causal inferences. Future longitudinal or experimental research could further explore the causal relationships between the constructs analyzed. Our research was conducted just prior to the COVID-19 pandemic. Nevertheless, studies during COVID-19 have shown that leadership practices in emergency departments are positively associated with structural and psychological empowerment of nurses [49], demonstrating the importance of nurse empowerment also in the new epidemiological context. Moreover, our research was conducted in public hospitals in Poland. Therefore, it seems advisable to conduct further studies among nurses working in different settings (e.g., outpatient clinics, hospices, and nursing homes) and in other countries to test the generalizability of our findings.

### 6.2. Implications for Nursing Management

Working under stress due to numerous demands, nurses are expected to be empathetic and sensitive to deal with situations such as costly recovery or non-recovery and death of patients [4]. This can lead to feelings of professional dissatisfaction, exhaustion, and disengagement from work. Therefore, remedies that can be implemented in healthcare organizations through leadership practices are important to reduce nurses' turnover intentions and improve quality of care. Leadership that creates empowering work conditions plays a fundamental role in creating supportive practice environments [10]. The present study shows that both structural and psychological empowerment can play an important role in creating such workplace conditions in hospitals. Our findings suggest that nursing and hospital management can enhance nurses' psychological empowerment through the development of structural empowerment practices.

Structural empowerment can be promoted through job design practices that include access to relevant information to perform one's job, effective feedback on performance and clear direction, and allocation of sufficient time for assigned tasks [11, 23]. An empowering work environment supports nurses by providing them with legitimate power and practicing transparency in management decisions [25], which may reduce their intention to leave [13]. Our findings show that empowering working conditions that ensure flexibility, time to perform professional duties, immediate help when needed, and appreciation from supervisors and colleagues are associated with higher job satisfaction among nurses. When trying to reduce job exhaustion, it is worthwhile to focus on creating a collaborative atmosphere in the department and cultivating nurses' partnership relationships with other hospital staff. It should be noted, however, that excessive demands related to continuous professional development may increase nurses' exhaustion. To promote psychological empowerment through structural empowerment, hospital managers also need to give nurses more autonomy in how they do their work, set clear goals, and promote teamwork and a cooperative atmosphere [11]. This can promote nurses' sense of efficacy, involvement in what is happening in the department, and a sense of being valued for their work. Open communication strategies, such as team briefings or a suggestion box, are also recommended to facilitate effective and transparent information sharing [11]. It is also worth noting that special programs have been developed, such as a psychodrama-based psychological empowerment program that increases psychological empowerment in nurses [50]. Thus, strategic planning for the professional development of health professionals can include the development of psychological empowerment in employees and the acquisition of competencies necessary for the implementation of structural empowerment practices by managers. All of these strategies are likely to promote structural empowerment and, through psychological empowerment, increase nurses' job satisfaction and their burnout and, consequently, reduce turnover [9].

## 7. Conclusions

The results of this study suggest that psychological empowerment is an underlying mechanism that may explain why structural empowerment at the hospital department level (i.e., organizational-level empowering work conditions) is positively related to job satisfaction and negatively related to burnout among nurses. This has implications for theory by extending the multilevel nomological network of the constructs examined and for management practice by highlighting the role of structural empowerment in work design in public health institutions.

## Figures and Tables

**Table 1:** Descriptive statistics, reliability, and correlations between variables.

Variable	M	SD	*α*	Correlation										
				1	1a	1b	1c	1d	1e	1f	2	3	4a	4b
(1) Structural empowerment	60.75	13.24	0.93	1										
(1a) Access to opportunity	11.06	2.59	0.81	0.71^*∗∗∗*^	1									
(1b) Access to information	9.19	3.23	0.91	0.77^*∗∗∗*^	0.48^*∗∗∗*^	1								
(1c) Access to support	10.25	2.85	0.91	0.82^*∗∗∗*^	0.56^*∗∗∗*^	0.63^*∗∗∗*^	1							
(1d) Access to resources	9.72	2.48	0.85	0.73^*∗∗∗*^	0.43^*∗∗∗*^	0.45^*∗∗∗*^	0.53^*∗∗∗*^	1						
(1e) Formal power	7.65	2.94	0.85	0.73^*∗∗∗*^	0.32^*∗∗∗*^	0.46^*∗∗∗*^	0.49^*∗∗∗*^	0.48^*∗∗∗*^	1					
(1f) Informal power	13.00	3.19	0.79	0.80^*∗∗∗*^	0.47^*∗∗∗*^	0.48^*∗∗∗*^	0.60^*∗∗∗*^	0.54^*∗∗∗*^	0.53^*∗∗∗*^	1				
(2) Psychological empowerment	59.46	10.65	0.89	0.55^*∗∗∗*^	0.36^*∗∗∗*^	0.41^*∗∗∗*^	0.36^*∗∗∗*^	0.42^*∗∗∗*^	0.47^*∗∗∗*^	0.48^*∗∗∗*^	1			
(3) Job satisfaction	21.05	6.01	0.86	0.59^*∗∗∗*^	0.39^*∗∗∗*^	0.41^*∗∗∗*^	0.46^*∗∗∗*^	0.48^*∗∗∗*^	0.36^*∗∗∗*^	0.56^*∗∗∗*^	0.49^*∗∗∗*^	1		
(4a) Burnout-exhaustion	20.94	3.93	0.61	−0.40^*∗∗∗*^	−0.21^*∗∗∗*^	−0.27^*∗∗∗*^	−0.23^*∗∗∗*^	−0.39^*∗∗∗*^	0.26^*∗∗∗*^	0.25^*∗∗∗*^	−0.35^*∗∗∗*^	−0.47^*∗∗∗*^	1	
(4b) Burnout-disengagement	19.51	3.23	0.77	−0.52^*∗∗∗*^	−0.39^*∗∗∗*^	−0.29^*∗∗∗*^	−0.40^*∗∗∗*^	−0.41^*∗∗∗*^	−0.39^*∗∗∗*^	−0.41^*∗∗∗*^	−0.41^*∗∗∗*^	−0.56^*∗∗∗*^	0.64^*∗∗∗*^	1

^*∗∗∗*^ *p* < 0.001.

**Table 2:** Results of multilevel modeling explaining psychological empowerment, job satisfaction, exhaustion, and disengagement from work.

Predictor (Level 2)	Dependent variable (Level 1)											
	Psychological empowerment			Job satisfaction			Burnout-exhaustion			Burnout-disengagement		
	*γ*	SE	*p*	*γ*	SE	*p*	*γ*	SE	*p*	*γ*	SE	*p*
*Model 1*:												
Structural empowerment	1.70	0.52	0.003	0.29	0.07	0.001	−0.11	0.05	0.040	−0.09	0.03	0.002

*Model 2*.												
Access to opportunity	0.05	0.93	0.954	0.26	0.33	0.429	0.82	0.39	0.046	−0.08	0.19	0.676
Access to information	0.97	0.74	0.201	−0.38	0.47	0.432	−0.63	0.45	0.176	−0.21	0.20	0.291
Access to support	−1.40	1.53	0.371	0.57	0.56	0.323	−0.93	0.53	0.094	−0.51	0.29	0.098
Access to resources	0.03	0.78	0.972	1.50	0.33	<0.001	−0.07	0.34	0.848	−0.06	0.23	0.783
Formal power	0.05	0.82	0.949	1.30	0.48	0.014	−0.85	0.56	0.145	−0.32	0.24	0.197
Informal power	2.23	1.44	0.135	−0.78	0.60	0.207	0.97	0.43	0.033	0.47	0.23	0.057

*γ* = unstandardized coefficient; SE = standard error.

**Table 3:** Results of cross-level mediation analyses: indirect effects explaining job satisfaction, exhaustion, and disengagement from work.

Indirect effect	95% CI
*Dependent variable: job satisfaction*	
Structural empowerment-psychological empowerment-job satisfaction	0.19, 0.79
Access to opportunity-psychological empowerment-job satisfaction	−0.51, 0.54
Access to information-psychological empowerment-job satisfaction	−0.13, 0.69
Access to support-psychological empowerment-job satisfaction	−1.26, 0.45
Access to resources-psychological empowerment-job satisfaction	−0.43, 0.45
Formal power-psychological empowerment-job satisfaction	−0.45, 0.47
Informal power-psychological empowerment-job satisfaction	−0.17, 1.45

*Dependent variable: burnout-exhaustion*	
Structural empowerment-psychological empowerment-burnout-exhaustion	−0.40, −0.08
Access to opportunity-psychological empowerment-burnout-exhaustion	−0.26, 0.24
Access to information-psychological empowerment-burnout-exhaustion	−0.33, 0.06
Access to support-psychological empowerment-burnout-exhaustion	−0.21, 0.61
Access to resources-psychological empowerment-burnout-exhaustion	−0.21, 0.21
Formal power-psychological empowerment-burnout-exhaustion	−0.26, 0.24
Informal power-psychological empowerment-burnout-exhaustion	−0.71, 0.08

*Dependent variable: burnout-disengagement*	
Structural empowerment-psychological empowerment-burnout-disengagement	−0.42, −0.09
Access to opportunity-psychological empowerment-burnout-disengagement	−0.27, 0.26
Access to information-psychological empowerment-burnout-disengagement	−0.36, 0.07
Access to support-psychological empowerment-burnout-disengagement	−0.23, 0.65
Access to resources-psychological empowerment-burnout-disengagement	−0.23, 0.22
Formal power-psychological empowerment-burnout-disengagement	−0.24, 0.23
Informal power-psychological empowerment-burnout-disengagement	−0.75, 0.08

CI = confidence interval for the indirect effect.

## Data Availability

The data used to support the findings of this study are available from the corresponding author upon request.
